# The Effect of Garcin® in Preventing AntiTB-Induced Hepatitis in Newly Diagnosed Tuberculosis Patients 

**Published:** 2014

**Authors:** Payam Tabarsi, Fanak Fahimi, Nader Heidarzadeh, Roodabeh Haghgoo, Mehdi Kazempour, Mohammadreza Masjedi, Ali Akbar Velayati

**Affiliations:** a *Clinical Tuberculosis and Epidemiology Research Center, National Research Institute of Tuberculosis and Lung Diseases (NRITLD), Shahid Beheshti University of Medical Sciences, Tehran, Iran*.; b *Clinical Pharmacy Department, School of Pharmacy, Shahid Beheshti University of Medical Sciences, Tehran, Iran.*; c *Chronic Respiratory Diseases Research Center, National Research Institute of Tuberculosis and Lung Diseases (NRITLD), Shahid Beheshti University of Medical Sciences, Tehran, Iran. *; d *Chemical Injuries Research Center, Baqiyatallah University of Medical sciences, Tehran, Iran. *; e *Mycobacteriology Research Center, National Research Institute of Tuberculosis and Lung Diseases (NRITLD), Shahid Beheshti University of Medical Sciences, Tehran, Iran.*; f *Telemedicine Research Center, National Research Institute of Tuberculosis and Lung Diseases (NRITLD), Shahid Beheshti University of Medical Sciences, Tehran, Iran. *

**Keywords:** Garlic, Tuberculosis, Drug-Induced hepatitis, Clinical trial

## Abstract

Adverse effects of antituberculosis agents such as hepatotoxicity may reduce treatment effectiveness, because they significantly contribute to nonadherence and eventually result in treatment failure, relapse or the emergence of drug resistance. Garlic is an ancient herbal substance, which its effectiveness on isoniazid and rifampicin-induced hepatic injury in animal models has been demonstrated (1).

In the present study a randomized, double blind, placebo-controlled, parallel group clinical trial was designed to assess the effect(s) of garlic tablets (1000 mg daily) administered for two weeks orally. Fifty eight newly diagnosed, smear positive pulmonary tuberculosis patients, with age ranges between 18-65 years old, were randomly allocated into two groups. Each patient received either garlic or placebo tablets for the first two weeks of tuberculosis treatment.

Of total 58 patients, 31 received garlic tablets while 27 received placebo. No significant difference was found between the two groups regarding age, sex, nationality, smoking, underlying diseases and opium usage. During 8 weeks of anti-TB (antituberculosis) treatment, 8 (13.0%) patients developed drug-induced hepatotoxicity (DIH). Of them, 6 (75%) occurred in the first two weeks of treatment. Fifty percent of the patients who developed DIH were in garlic group. Results indicated no significant difference between groups in developing DIH (*p*=1.000).

We could not show a significant role in preventing DIH by 1000 mg daily garlic administration.

## Introduction

Tuberculosis (TB) is one of the principal reasons of mortality from an infectious disease. About 8.6 million new TB cases were reported in 2012 and 1.3 million of them died (2). Hepatic damage is the most serious adverse effect of antituberculosis treatment and has a high rate in some developing countries such as Iran, where the reported rates vary from 4.9% to 27.7% (3, 4, 5). The incidence of 13% was indicated at the national TB referral center (6).

Isoniazid (INH), rifampicin (RIF) and pyrazinamide are potentially hepatotoxic drugs. Antituberculosis drug-induced hepatotoxicity (DIH) causes considerable morbidity and mortality and diminishes treatment effectiveness. It significantly adds on issues such as non adherence, treatment failure, relapse or the emergence of drug resistance (7). It frequently requires treatment modification, which may have negative consequences for treatment outcome. Asymptomatic liver enzymes elevation is common during antituberculosis treatment, but hepatotoxicity can be fatal when not recognized in early stages (8).

Studies have shown that INH-RIF-induced oxidative injury can be prevented by supporting the cellular antioxidant defense mechanism by using agents with antioxidant properties such as N-acetylcysteine (9). Freshly prepared garlic homogenate, which has an antioxident effect, has also been shown to have a protective effect against INH+RIF-induced liver injury in animal models (1, 10). However, there are no published data regarding the protective effect of garlic against antituberculosis DIH in humans, if any, to our knowledge. 

Hence, this clinical trial was designed to study the effect of garlic on DIH in newly diagnosed tuberculosis patients.

## Experimental

*Setting*

The study was carried out at Masih Daneshvari hospital from March to September 2009. This tertiary center is the sole national referral hospital for TB, holds the national reference laboratory, comprises the National Research Institute of Tuberculosis and Lung Disease (NRITLD), Tehran, Iran and serves as the WHO collaborating center in the Middle East region(6).

*Case recruitment*

Written informed consent was obtained from all of the participants.

Fifty eight inpatients, 18-65 years old, who were newly diagnosed with smear positive pulmonary tuberculosis completed the study. They all received standard antituberculosis regimen. 

Patients with positive HIV, confirmed hepatitis, cirrhosis, renal failure and known hypersensitivity to garlic and those prone to hemorrhage and/or hypotension were excluded.

*Treatment and monitoring*

A randomized, double blind, placebo-controlled, parallel group, clinical trial was designed to assess the treatment effects of garlic tablets administered for two weeks orally. 

This clinical trial was registered in the Australian New Zealand Clinical Trial Registry, and the registry number is ACTRN12609000703202.

Patients were randomly allocated into two groups. The treatment allocation was randomized by a researcher who was not directly involved in the trial and computerized method was used to generate the sequence in which subjects would be randomized. The treatment group received 2 tablets (1000 mg) of Garcin (Gol Daru, Isfahan, Iran) per day for the first 2 weeks of the standard anti-tuberculosis regimen. For the placebo group, identical lactose pills manufactured by the same company were administered daily for the same period of time. Since the placebo pills lack the smell of the real drug, the treatment tablets were put in the vicinity of the plastic bags for a week and then the bags were filled by placebo. 

Patients’ sputum smears were monitored at baseline, 4 and 8 weeks. Also the clinical symptoms and liver function tests including Serum Glutamic Oxaloacetic Transaminase (SGOT), Serum Glutamic Pyruvic Transaminase (SGPT), and alkaline phosphatase were monitored at baseline, 2, 4 and 8 weeks due to the fact that drug-induced hepatotoxicity is considered when alanine amino transferase levels are increased up to three times the upper limit of normal (ULN) in the presence of hepatitis symptoms or elevation of up to five times the ULN in the absence of symptoms (11).

*Statistical analysis*

Fisher’s exact test was used to assess the effects of garlic on reducing the rate of antituberculosis DIH compared to the placebo. 

To describe the relationship between the outcome and the set of independent variables logistic regression model was used but due to limited sample size, the achieved model was not satisfactory. p*-*values ≤0.05 was used as significance level.

## Results

**Figure 1:**
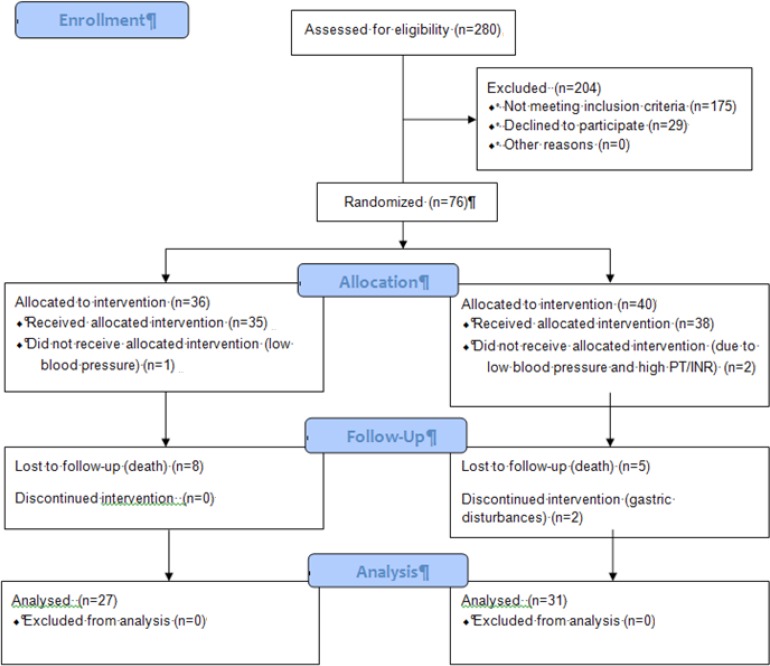
Flow Diagram of the patients

Figure 1. represents the flow diagram of our patients. Fifty eight new cases of tuberculosis were included and analysed in the study. Patients’ characteristics and clinical factors are summarized in Table 1. Of the total 58 patients who completed the study protocol, 31 received garlic tablets while other 27 received placebo. 

**Table 1:** Patients’ characteristics and clinical factors

**Patients’ characteristics**	**Total patients (%)**	**Treatment group (%)**	**Placebo group (%)**	**p-Value **
Sex				
Male Female	21(36.2) 37(63.8)	11(35.5) 20(64.5)	10(37) 17(63)	NS
Age				
Range Median	17-70 35.53	17-63 37.25	18-70 35.55	NS
Nationality				
Iranian Afghan	34(58.6) 24(41.4)	17(54.8) 14(45.2)	17(63) 10(37)	NS
Patient class				
Smear positive Other	21(36.2) 37(63.8)	12(38.7) 19(61.3)	9(33.3) 18(66.7)	NS
Underlying disease				
Diabetes Mellitus Chronic Renal Failure Ischemic Heart Disease Other	7(12.1) 1(1.8) 2(3.6) 12(20.7)	3(9.7) 0(0) 2(6.4) 4(12.9)	4(14.8) 1(3.7) 0(0) 8(29.6)	NS
Smoking				
Active smoker Passive smoker None	9(15.5) 1(1.8) 48(82.8)	4(12.9) 1(3.2) 26(83.9)	5(18.5) 0(0) 22(81.5)	NS
Opium inhalation				
Yes No	8(13.8) 50(86.2)	4(12.9) 27(87.1)	4(14.8) 23(85.2)	NS
Drug-induced hepatitis	8(13.8)	4(12.9)	4(14.8)	NS
Hepatitis follow-up				
2nd week follow-up 4th week follow-up 8th week follow-up	6(75) 1(12.5) 1(12.5)	3(9.7) 0(0) 1(3.2)	3(11.1) 1(3.7) 0(0)	NS
Outcome				
Under treatment Cured Failure Complete treatment	25(43.1) 21(36.2) 3(5.2) 9(15.5)	16(51.6) 10(32.3) 1(3.2) 4(12.9)	9(33.3) 11(40.7) 2(7.4) 5(18.5)	NS
Total	58	31	27	

Males comprised 21 (36.2%) of the cases. Thirty four (58.6%) of the patients were Iranian and the remaining 24 (41.4%) were Afghan. Nine (15.5%) patients were active smokers while 48 (82.8%) were non smokers and one was passive smoker. No significant difference was found between groups regarding age, sex, nationality, smoking, underlying diseases and opium addiction. During 8 weeks of anti-TB treatment, 8 (13.0%) patients developed drug-induced hepatitis (DIH), 6 (75%) of them did so in the first two weeks of treatment, 1 (12.5%) between two and four weeks and 1 (12.5%) between four and eight weeks from the initiation of the treatment.

Four of 8 (50%) patients who developed DIH were in the treatment group. There was no significant difference between patients with hepatitis and without hepatitis considering variables *i.e*. age, sex, nationality, smoking, underlying diseases and opium inhalation (Table 2).

Results indicated no significant difference between the two groups in developing DIH (*p*=1.000).

**Table 2:** Patients’ characteristics due to developing DIH

**Patients’ characteristics**	**Drug-induced hepatitis (%)**	**Without hepatitis (%)**	**p-Value**
Sex			
Male Female	3(37.5) 5(62.5)	18(36) 32(64)	NS
Age			
Range Median	17-51 34.5	17-70 35.7	NS
Nationality			
Iranian Afghan	4(50) 4(50)	30(60) 20(40)	NS
Patient class			
Smear positive Other	4(50) 4(50)	17(34) 33(66)	NS
Underlying disease			
Diabetes Mellitus Chronic Renal Failure Ischemic Heart Disease Other	1(12.5) 0(0) 0(0) 3(37.5)	6(12) 1(2) 2(4) 9(18)	NS
Smoking			
Active smoker Passive smoker None	0(0) 1(12.5) 7(87.5)	9(18) 0(0) 41(82)	NS
Opium inhalation			
Yes No	0(0) 8(100)	8(16) 42(84)	NS
Outcome			
Under treatment Cured Failure Complete treatment	4(50) 3(37.5) 1(12.5) 0(0)	21(42) 18(36) 2(4) 9(18)	NS
Total	8	50	

## Discussion

In the present study the probable protective effect of garlic on decreasing the drug-induced hepatotoxicity in newly diagnosed tuberculosis inpatients was investigated. 

Patients received garlic or placebo for 2 weeks from the initiation of the treatment. All the patients were precisely monitored regarding clinical and laboratory parameters for the first two months of treatment. As it was presumed 6 out of 8 patients developed drug-induced hepatotoxicity during the first 2 weeks. Studies indicated that DIH most often occurs during the first 2 weeks of anti-TB treatment (6). No significant difference was shown regarding variables (age, sex, nationality, smoking, underlying diseases and opium inhalation using analytical methods), between patients who developed DIH and those who did not four of 8 patients who developed DIH were in garlic group. Statistical analysis indicated no significant difference between the treatment group and placebo group in developing antituberculosis DIH despite receiving garlic for two weeks. Consequently, our study could not confirm the protective effect of garlic administration against DIH, however, a variety of antioxidants are found in garlic, which may make this drug eligible to prevent disease-causing oxidative harm (12). Metabolism pathways and toxic metabolites of anti-TB agents play a central role in hepatotoxicity of these agents (13). Garlic and the related organosulfur compounds have antioxidant and detoxifying properties. These detoxifying effects are due to their inhibiting effect on phase I enzymes and induction of phase II enzymes via binding to exogenous toxins through sulfhydryl groups (12). 

Garlic can inhibit lipid peroxidases. Garlic beneficial effect in saving the liver from substance induced damage may be through its additional advantages of lowering the plasma lipids (14). While we were unable to show any clinical effect of the drug in our patient population. 

Previous studies on the mechanism of INH+RIF-induced hepatotoxicity have shown that non-protein thiols play a crucial role in the detoxification of reactive toxic metabolites. Liver injury is more prevalent when glutathione stores are noticeably depleted. It has been indicated that garlic involves in modulation of the glutathione-related antioxidant system. An increase in glutathione s-transferase activity and a decrease in glutathione peroxidase activity were noted in garlic-treated rats (1, 15). 

Non-protein thiol is considered a defense mechanism in living cells. It protects cellular elements from peroxidase formed during metabolism and other reactive oxygen species reactions, since it acts as a substrate for antioxidant enzymes *i.e*. glutathione peroxidase and glutathione reductase. Garlic extract increases cellular glutathione in a variety of cells including those in liver (16). 

Our inability in showing its positive impact, if any, on reducing the rate of hepatitis may be due to different reasons. Our study population was limited in size. In spite of the fact that the rate of hepatitis in our studied patients was almost the same as previously reported studies, the number of the patients with DIH (8 totally) may be insufficient to demonstrate the likely impact. Also the dose and duration of the treatment should be reevaluated for future researches.

## References

[1] Pal R, Vaiphei K, Sikander A, Singh K, Rana S (2006). Effect of garlic on isoniazid and rifampicin-induced hepatic injury in rats. World J. Gastroenterol.

[2] World Health Organization Tuberculosis. http://www.who.int/mediacentre/factsheets/fs104/en/.

[3] Ghasemi Barghi R, Haj Agha Mohammadi AA, Samimi R (2011). Drug-induced hepatitis (Abundance and outcome during course of tuberculosis treatment): Seven-year Study on 324 patients with positive sputum in Iran. Govaresh.

[4] Sharifi-mood B, Kouhpayeh HR, Salehi M (2006). Incidence of hepatotoxicity due to antitubercular medicine and assessment of risk factors, Zahedan, Iran. J. Med. Sci.

[5] Sharifzadeh M, Rasoulinejad M, Valipour F, Nouraie M, Vaziri S (2005). Evaluation of patient-related factors associated with causality, preventability, predictability and severity of hepatotoxicity during antituberculosis treatment. Pharmacol. Res.

[6] Baghaei P, Tabarsi P, Chitsaz E, Saleh M, Marjani M, Shemirani Sh, Pooramiri M, Kazempour M, Farnia P, Fahimi F, Mansouri D, Masjedi M (2010). Incidence, Clinical and Epidemiological Risk Factors and Outcome of Drug-Induced Hepatitis due to antituberculous Agents in New Tuberculosis cases. Am. J. Ther.

[7] Yee D, Valiquette C, Pelletier M, Parisien I, Rocher I, Menzies D (2003). Incidence of serious side effects from first-line antituberculosis drugs among patients treated for active tuberculosis. Am. J. Respir. Crit. Care Med.

[8] Tostmann A, Boeree MJ, Aarnoutse RE, de Lange WC, van der Ven AJ, Dekhuijzen R (2008). Antituberculosis drug-induced hepatotoxicity: Concise up-to-date review. J. Gastroenterol. Hepatol.

[9] Baniasadi Sh, Eftekhari P, Tabarsi P, Fahimi F, Raoufy MR, Masjedi MR, Velayati AA (2010). Protective effect of N-acetylcysteine on antituberculosis drug-induced hepatotoxicity. Eur. J. Gastroen. Hepat.

[10] Meghna R, Adhvaryu MR, Reddy N, Parabia MH (2007). Effects of four Indian medicinal herbs on Isoniazid-, Rifampicin- and Pyrazinamide-induced hepatic injury and immunosuppression in guinea pigs. World J. Gastroenterol.

[11] Kaplowitz N (2004). Drug-induced liver injury. Clin. Infect. Dis.

[12] Munday R, Munday CM (2004). Induction of phase II enzymes by aliphatic sulfides derived from garlic and onions: an overview. Methods Enzymol.

[13] Sistanizad M, Azizi E, Khalili H, Hajiabdolbaghi M, Gholami Kh, Mahjub R (2011). Anti tuberculosis druinduced hepatotoxicity in Iranian tuberculosis patients: Role of Isoniazide metabolic polymorphism. Iran. J. Pharm. Res.

[14] Park EY, Ki SH, Ko MS, Kim CW, Lee MH, Lee YS, Kim SG (2005). Garlic oil and DDB, comprised in a pharmaceutical composition for the treatment of patients with viral hepatitis, prevents acute liver injuries potentiated by glutathione deficiency in rats. Chem. Biol. Interact.

[15] Wu CC, Sheen LY, Chen HW, Tsai SJ, Lii CK (2001). Effects of organosulfur compounds from garlic oil on the antioxidation system in rat liver and red blood cells. Food and Chemical Toxicol.

[16] Tanaka S, Haruma K, Kunihiro M, Nagata S, Kitadai Y, Manabe N, Sumii M, Yoshihara M, Kajiyama G, Chayama K (2004). Effects of aged garlic extract (AGE) on colorectal adenomas: a double-blinded study. Hiroshima J. Med. Sci.

